# The territorial nature of aggression in biofilms

**DOI:** 10.3389/fmicb.2022.878223

**Published:** 2022-08-23

**Authors:** Ihab Hashem, Jan F. M. Van Impe

**Affiliations:** Department of Chemical Engineering, KU Leuven, Ghent, Belgium

**Keywords:** evolutionary game theory (EGT), aggression, biofilms, bacteriocins, individual based modeling

## Abstract

Microbial conflicts have a particularly aggressive nature. In addition to other chemical, mechanical, and biological weapons in their repertoire, bacteria have evolved bacteriocins, which are narrow-spectrum toxins that kill closely related strains. Bacterial cells are known to frequently use their arsenal while competing against each other for nutrients and space. This stands in contrast with the animal world, where conflicts over resources and mating opportunities are far less lethal, and get commonly resolved *via* ritualized fighting or “limited war” tactics. Prevalence of aggression in microbial communities is usually explained as due to their limited ability to resolve conflicts *via* signaling as well as their limited ability to pull out from conflicts due to the sessile nature of their life within biofilms. We use an approach that combines Evolutionary Game Theory (EGT) and Individual-based Modeling (IbM) to investigate the origins of aggression in microbial conflicts. In order to understand how the spatial mode of growth affects the cost of a fight, we compare the growth dynamics emerging from engaging in aggression in a well-mixed system to a spatially structured system. To this end, a mathematical model is constructed for the competition between two bacterial strains where each strain produces a diffusible toxin to which the other strain is sensitive. It is observed that in the biofilm growth mode, starting from a mixed layer of two strains, mutual aggression gives rise to an exceedingly high level of spatial segregation, which in turn reduces the cost of aggression on both strains compared to when the same competition occurs in a well-mixed culture. Another observation is that the transition from a mixed layer to segregated growth is characterized by a switch in the overall growth dynamics. An increased “lag time” is observed in the overall population growth curve that is associated with the earlier stages of growth, when each strain is still experiencing the inhibiting effect of the toxin produced by its competitor. Afterwards, an exponential phase of growth kicks in once the competing strains start segregating from each other. The emerging “lag time” arises from the spiteful interactions between the two strains rather than acclimation of cells' internal physiology. Our analysis highlights the territorial nature of microbial conflicts as the key driver to their elevated levels of aggression as it increases the benefit-to-cost ratio of participating in antagonistic interactions.

## 1. Introduction

One aspect in which bacteria drastically differ from free roaming animals is the level of aggression in the microbial world (Granato et al., 2019). Animals, from the same species, generally avoid engaging in lethal fights among each other for resources, territory or access to mates (Eibl-Eibesfeldt, 1961; Smith and Price, 1973). One example of such “ritualized fighting” is how snakes avoid using their venom against each other and opt instead for wrestling contests to settle their conflicts (Shaw, 1948). On the other hand, bacteria have evolved bacteriocins, which are narrow spectrum toxins that aim to inhibit the growth of closely related strains (Gardner et al., 2004; Riley and Chavan, 2006; Hibbing et al., 2010; Alvarez-Sieiro et al., 2016; Granato et al., 2019). Additionally, many bacterial species enjoy the capacity of producing antibiotics, broad spectrum toxins which aim to kill distantly related species (Kohanski et al., 2010; Ageitos et al., 2017). That is in addition to other mechanical (Gebhart et al., 2012) and even biological weapons in their arsenal (Brown et al., 2009; Granato et al., 2019).

What drives the evolution of a high level of aggression in the microbial world? A possible answer is that bacteria have a limited capacity to resolve their conflicts *via* signaling (Granato et al., 2019). When two animals face-off in a conflict, they can exchange different kinds of signals to communicate their relative strengths, and consequently, avoid a costly fight (Maynard-Smith et al., 2003; van Lieshout et al., 2005). Bacterial regulatory networks are clearly more simple than animal brains, and hence are subject to more constraints to evolve such signaling systems to resolve conflicts. Also, another reason that is usually invoked to explain the lethal nature of bacterial contests is the sessile nature of their life, an aspect they share with plants and fungi (Sestari and Campos, 2021). Bacteria commonly grow as surface attached communities of cells, known as biofilms, enclosed in a self-produced extracellular polymeric matrix. Hence, most cells inside the biofilm are effectively sessile, which translates into a limited ability to run away from conflicts compared to a free roaming animal (Granato et al., 2019; Rillich and Stevenson, 2019).

The fundamental difference in nature between microbial and animal contests, sessile vs. motile, has resulted in a corresponding difference in the mathematical frameworks used to tackle each problem. Models of animal contests that do not take space into account, such as the classical paper by Smith and Price (1973), have been used to explain animal behaviors in contests. On the other hand, the evolution of social phenotypes in biofilms has been successfully studied by a spatial modeling framework, called Individual-based modeling (IbM) (Kreft et al., 2001). In IbM, the individual cell behavior is explicitly modeled, by describing all its main known physiological processes such as its growth, reproduction, death, and motility, while the spatial structure of the environment is also modeled with a focus on diffusion phenomena. Then, the simulation gets seeded with a number of cells and the population behavior emerges from the interactions between individual agents. IbMs have been applied to explain a wide range of social phenomena in biofilms, including aspects of cooperation (Kreft, 2004; Mitri et al., 2011), competition (Xavier and Foster, 2007; Bucci et al., 2011), and communication (Schluter et al., 2016).

Smith and Price (1973) tackled the problem of explaining limited wars in animal conflicts *via* the construction of the influential hawk-dove model. They managed to prove that the hawk, which is an abstraction of an aggressive strategy, will not necessarily dominate the population. The evolutionary stable state of the population has been shown to be a polymorphic state with both hawks and doves. And the level of aggression in the population, the fraction of the population that displays a hawk behavior at equilibrium, has been shown to be proportional to the ratio between the value of the resource in dispute to the cost of a potential fight. This result has been later verified experimentally (Hansen, 1986; Tainaka et al., 2007; Oprea et al., 2011) and it transcends the assumptions of this particular model to even political conflicts (Georgiev et al., 2013; Glowacki et al., 2017; Krahé, 2020). A striking example is the difference between conflict patterns among carnivores and herbivorous animals (Georgiev et al., 2013). Animals which compete for high nutritional value resources, like carnivores, undergo contests that are characterized by having high resource value to cost ratio, as the food is scarce and relatively nutritious. Hence, relatively violent fights erupt (Kruuk and Kruuk, 1972; Wrangham, 1980; Koenig and Borries, 2009). On the other hand, for herbivorous species, since grass has low nutritional value, the value of the resource to cost of a fight ratio is relatively small. And these animals hence tend to avoid direct, aggressive, contests (Isbell, 1991; Young and Isbell, 2002).

In the microbial world, aggression, or spite, has been found to be dependent on the scale of competition (Gardner and West, 2004), where bacteriocin production has been found to be most favored when the competition is localized and at intermediate relatedness (Gardner et al., 2004). Czárán et al. (2002) and Weber et al. (2014) analyzed how spiteful interactions help to maintain and promote microbial diversity. Using a spatial game theoretic model, Czárán et al. (2002) showed how diversity can be maintained in a three strain system consisting of an antibiotic producer, resistant, and sensitive strains. Further research focused on the selection pressures driving the evolution of toxin regulation (Doekes et al., 2019) as well as the role of stochastic processes in this process (von Bronk et al., 2017). It is also noted that bacteriocin production by bacteria is a cooperative behavior from the point of view of the clonemates. The phenomenon of cheating in the context of bacteriocin production has been studied by West et al. (2006) and Rankin et al. (2007).

In this paper, we investigate the aggression dynamics in microbial conflicts, with a focus on how the spatial mode of microbial growth as in biofilms affects the cost of engaging in aggression endured by a microbial strain. To this end, a model is constructed of the competition between two strains, where each strain is capable of producing a bacteriocin to which the other strain is sensitive. Different aggression scenarios are investigated in both models of well-mixed cultures as well as of a spatially structured environment, growing as a biofilm, using EGT and IbM (Hashem and Van Impe, 2022a,b). An EGT analysis is carried out first to find when engaging in aggression is a beneficial strategy in the well-mixed culture, as well as the optimal level of metabolic investment in bacteriocin production in such case. Afterwards, using an IbM of biofilm growth, the cost of engaging in aggression expressed as the suppression of microbial growth rate as well as the growth dynamics of the two-strain community are investigated, with a focus on the relationship between spatial structuring and engaging in aggression.

## 2. Model description

### 2.1. Spatially mixed growth

**Figure 1:**
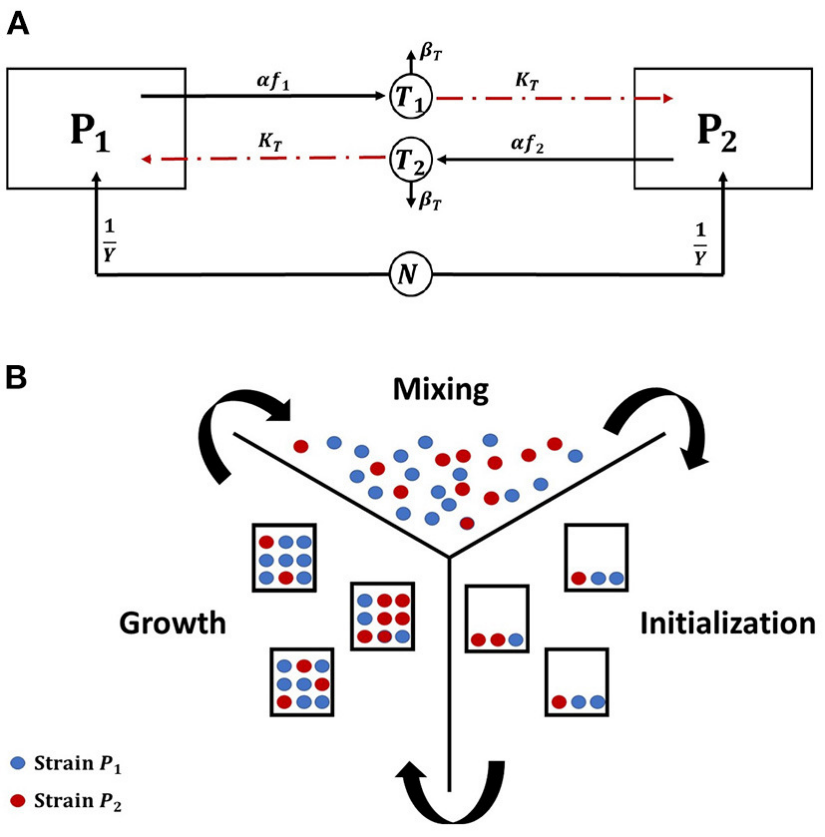
**(A)** An illustrative diagram (Hashem and Van Impe, 2022a,b) of a competition between two constitutive toxin producers, where each strain is immune to its own toxin, growing together on the same nutrient. The diagram depicts two biological species, *P*_1_ and *P*_2_, each denoted by a square, and three chemical species, *N*, *T*_1_, and *T*_2_, each denoted by a circle and representing the common nutrient and the toxins produced by *P*_1_ and *P*_2_, respectively. The consumption of *N* as well as the production of *T*_1_ and *T*_2_ are denoted by solid lines, while the inhibiting effects of *T*_1_ and *T*_2_ on *P*_2_ and *P*_1_, respectively, are denoted by dashed lines. **(B)** An illustration of the microbial life cycle model: a metapopulation of the two strains *P*_1_ and *P*_2_ is assumed to grow in a finite number of separate patches. The model consists of (i) an initialization step: all patches are seeded with the bacterial strains, (ii) a growth step: the growth dynamics is simulated in each patch separately until the population levels of all strains reach a steady state, and (iii) a mixing step: all patches are mixed with each other and the new composition of the metapopulation is used to initialize a new cycle of the model.

A model of two bacterial strains, each capable of constitutively producing its own toxin and both competing over the same nutrient is constructed. Each strain is assumed to be immune to its own toxin while being vulnerable to the opponent's toxin. The two strains are assumed to have identical growth parameters, and similar sensitivity to the toxin produced by the other strain. The model is described by the following set of equations, with the model's parameters provided in Table 1 (Bucci et al., 2011; Cornforth and Foster, 2013):


(1) $$\begin{array}{c} \frac{dP_{1}}{dt}=\left(\left(1-f_{1}\right)\mu -K_{T}T_{2}\right)P_{1} \end{array}$$

(2) $$\begin{array}{c} \frac{dP_{2}}{dt}=\left(\left(1-f_{2}\right)\mu -K_{T}T_{1}\right)P_{2} \end{array}$$

(3) $$\begin{array}{c} \frac{dT_{1}}{dt}=\alpha f_{1}\mu P_{1}-\beta_{T}T_{1} \end{array}$$

(4) $$\begin{array}{c} \frac{dT_{2}}{dt}=\alpha f_{2}\mu P_{2}-\beta_{T}T_{2} \end{array}$$

(5) $$\begin{array}{c} \frac{dN}{dt}=\frac{-1}{Y}\mu \left(P_{1}+P_{2}\right) \end{array}$$

(6) $$\begin{array}{c} \mu =\mu_{max}\frac{N}{N+K_{N}} \end{array}$$

with *P*_1_ and *P*_2_ as the biomass densities of the two bacterial strains (mg bacteria l^-1^). The concentrations of the two toxins (mg/l) are denoted by *T*_1_ and *T*_2_, respectively. *f*_1_ and *f*_2_ is the fraction of growth energy invested in producing the toxin by *P*_1_ and *P*_2_, respectively. *K*_*T*_ and β_*T*_ are the killing rate (l mg toxin^-1^ h^-1^) and the decay rate (h^-1^) of both toxins. *N* is the nutrient concentration. μ and *Y* are the growth rate (h^-1^) and mass yield (mg bacteria mg nutrients^-1^) of both strains, respectively. The growth equation is modeled using Monod kinetics with μ_*max*_ and *K*_*N*_ as the maximum specific growth rate (h^-1^) and the half saturation constant (mg l^-1^), respectively. The model is visually represented in Figure 1A.

**Table 1:** Model parameters.

**Parameter**	**Denotation**	**Value**
*f*	Fraction of energy invested in toxin production	0.1
*K*_*N*_	Half saturation constant	5 × 10^−4^ (mg l^-1^)
*K*_*T*_	Toxin's killing rate	9 × 10^−4^ (l mg toxin^-1^ h^-1^)
β_*T*_	Toxin's decay rate	10^−1^ (h^-1^)
μ_*max*_	Maximum growth rate	1 (h^-1^)
*Y*	Growth yield	0.7 (mg bacteria/ mg nutrients^-1^)
α	Toxin's stiochiometric coefficient	4 (mg toxin/ mg bacteria^-1^)
*D*_*S*_	Substrate's diffusivity	4 × 10^4^ (μm^2^ h^-1^)
*D*_*T*_	Toxin's diffusivity	4 × 10^4^ (μm^2^ h^-1^)

### 2.2. Invasion analysis

To find the optimal level of metabolic investment that should be allocated by any of the two strains in toxin production, a technique from EGT, invasion analysis, is used. Here, it is assumed that the two strains initially have the same level of metabolic investment in toxin production, *f*_*res*_, termed here the resident strategy. Then, a rare mutant appears in the population with different level of investment, *f*_*mut*_, that may or may not be able to invade the population and displace the resident strategy. The aim of the analysis is to find the resident strategy that if adopted by the whole population can not be displaced by any mutant strategy. To do that, the concepts of the microbial life cycle from Cremer et al. (2012) and the invasion index from Mitri et al. (2011) and Nadell et al. (2010) have been used (Niehus, 2016). The standard microbial life cycle is a modeling approach which aims to mirror the cycle of microbial growth in nature. The growth of bacteria involves the processes of colonies initiation, maturation and then dispersal, to form new colonies. So, in this modeling paradigm, bacteria are assumed to grow at separate patches, that later undergo a mixing step. In this mixing step, cells from all patches are assumed to undergo dispersal and get mixed with each other to initiate new colonies and start the next cycle of the simulation (Niehus, 2016; Niehus et al., 2021). Previous work (Gardner and West, 2004; Bucci et al., 2011) has already shown that aggression is favored when the competition between strains is more localized. Our work focuses on the effect of the spatial nature of growth in biofilms on the evolution of aggression. And a high level of mixing between patches is assumed. The model, illustrated in Figure 1B, begins with an initialization step: the patches are seeded with the bacterial strains, then a growth step: the growth of bacteria is simulated at each patch separately, until the populations of all strains reach a steady state. And finally a mixing step: the total mass of each strain over all patches is computed so that it can be accordingly redistributed over the patches in the next simulation cycle.

The evolutionary stable toxin production strategy, represented by f^*^, is the investment in toxin production that, if it is adopted by the population, no mutant with a different *f* can invade the population. Instead of explicitly modeling the microbial life cycle, the invasion analysis is carried out by calculating an invasion index (Mitri et al., 2011). The implicit assumption of the invasion analysis is that a mutant strategy, *f*_*mut*_, is set to spread in the population if the fitness of the mutant against a strain adopting the resident strategy, Π(*f*_*mut*_∣*f*_*res*_), is higher than the average fitness of the population. The fitness of any strain here is equivalent to its biomass by the end of the simulation when the population levels reach a steady state and the nutrient is completely consumed. The average fitness of the population is defined as the fitness of any of the two strains adopting the resident strategy against each other, Π(*f*_*res*_∣*f*_*res*_). Hence, a mutant toxin production strategy is set to invade a population adopting a resident toxin production strategy whenever the invasion index, *I*_*inv*_, is higher than one, where:


(7) $$\begin{array}{c} I_{inv}=\frac{\text{Π}\left(f_{mut}|f_{res}\right)}{\Pi \left(f_{res}|f_{res}\right)} \end{array}$$

An Evolutionary Stable Strategy (ESS), f^*^, is one that can not be invaded by any mutant strategy. To determine it, the invasion analysis is repeatedly carried out where the invasion indices of all possible mutant strategies are calculated against all possible resident strategies. The results are plotted in pairwise invasibility plots (Brännström et al., 2013), which show when a mutant strategy can invade a resident strategy. A resident strategy that can not be invaded by any mutant strategy is set to be f^*^. All the simulations were solved in MATLAB using ODE45 solver, where the initial biomass density for both species is set to be 1 mg bacteria *l*^−1^ and the initial nutrient concentration is 10^4^*mgl*^−1^. The running time is set to be 500 h, at which the nutrient is completely consumed in all simulations.

### 2.3. Biofilm modeling

For modeling competition within a biofilm, IbM simulations were carried out using MICRODIMS, an in-house IbM platform that has been built and applied to simulate microbial growth on surfaces as biofilms or colonies (Verhulst et al., 2011; Tack et al., 2015, 2017). MICRODIMS is implemented in Repast Simphony toolkit (North et al., 2013), an open-source individual based modeling toolkit, written in Java. It shares the same design principles of other established microbial IbM tools that have been used to study the role played by spatial growth on the social interactions in a microbial community (Picioreanu et al., 1998; Kreft et al., 2001; Xavier and Foster, 2007; Mitri et al., 2011). The models simulate the growth of cells on an inert surface. Cells are modeled as individual agents that consume nutrients, grow, reproduce, and die, with nutrients diffusing from an infinite source through the upper boundary of the simulation. Hence, Dirichlet boundary conditions are applied such that the chemicals' concentrations are held at their initial value at the upper boundary of the environment, while Neumann boundary conditions are applied at the solid surface such that the concentration gradients at the surface are equal to zero (Tack et al., 2015). Periodic boundary conditions are applied at the lateral ends of the model. The diffusion of nutrients and toxins in the model is solved using a Forward-Time-Central-Space algorithm (Anderson et al., 1997). A shoving algorithm (Kreft et al., 2001) is used to avoid the overlapping of cells. A detailed overview for the processes as well as the parameter values describing the movement, growth, reproduction and death of cells can be found in Tack et al. (2015). All the simulations were carried out using 50 replicates, and the mean of the results and their standard deviation have been plotted, with the standard deviation ≈ 1%, which is equivalent to a confidence interval >95%. And, unless otherwise is mentioned, they were conducted using a 800 × 250 μm grid, seeded with a mixed layer consisting of 80 cells of each strain and carried out till the biofilm height reached 150 μm. The fitness of each species is described in terms of its average growth rate, its total biomass divided by the time required to reach the maximum biofilm height at the end of the simulation. The initial nutrient concentration is set to be 10^4^ mg/l throughout all simulations. The high initial nutrient concentration as well as starting with a dense mixed initial layer of cells serve to minimize the effect of spatial structuring that emerge from competition over nutrients.

## 3. Results and discussion

### 3.1. Competition dynamics in a well-mixed system

**Figure 2:**
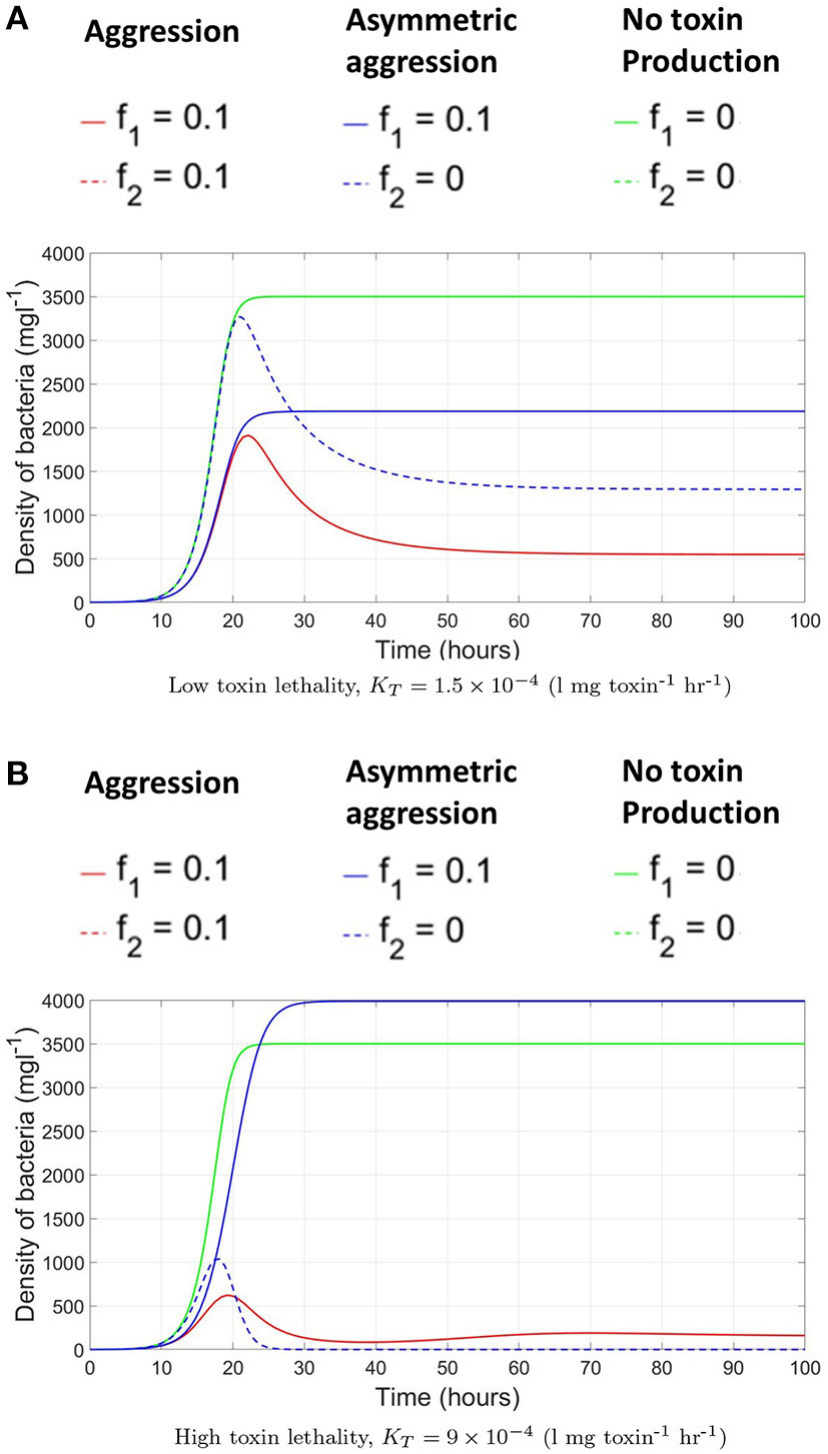
The evolution of the biomass density of the two bacterial strains in time, under the scenarios of (i) symmetric aggression (red): both strains produce toxins, by setting the toxin investment fraction, *f*, for both of them to 0.1 (ii) asymmetric aggression (blue): one of the strains, *P*_1_, produce a toxin, *f* = 0.1, while the other fully invests in growth, *f* = 0 and (iii) No toxin production (green): the two strains do not produce toxins and both fully invest in growth, *f* = 0 for both strains. **(A)** At low toxin lethality. **(B)** At high toxin lethality.

We investigate the conditions under which toxin production is a useful strategy in the context of the competition between two toxin producing strains in a well mixed culture, where all cells experience the same chemicals' concentrations. It is expected that releasing toxin would not always be an advantageous growth strategy. For example, if the toxin killing rate, *K*_*T*_, is not high enough, then a bacterial strain would fare better by not engaging in aggression. Also, when the nutrients in the system are scarce, it would be better for the microbes to allocate all their metabolic energy to biomass production. An example of how the model's parameters can affect when aggression becomes a favorable strategy is illustrated in Figure 2. Here, three contest scenarios are simulated: (i) symmetric aggression: both strains adopt an aggressive growth strategy, by producing toxin at a fraction of investment corresponding to 10% of their growth rates, consequently inhibiting each other's growth, (ii) asymmetric aggression: one strain is investing in toxin production while the other is not, and (iii) no toxin production: the two strains do not produce toxins and opt instead to fully allocate their resources to growth. The biomass-time evolution of the two strains is tracked for each of the three different scenarios, where the fitness of each strain is defined as its final biomass by the end of the simulation. When the toxin killing rate is low, we find that no toxin production becomes the ESS for both strains. As seen in Figure 2A, for the focal strain *P*_1_, the no toxin production strategy strictly dominates the toxin production strategy regardless of the behavior of the opposing strain. While an aggressive strain can outgrow a non toxin producing strain in the asymmetric scenario, switching to no toxin production growth strategy leads to a higher final biomass regardless to whether the opponent produces toxin or not. It is noted here that not producing toxin becomes an optimal growth strategy under the model's assumption of high mixing between patches. If the competition is more localized, as explained in Gardner and West (2004), then it would suffice for a toxin producer to outgrow a non toxin producer locally, as in the mixed case in Figure 2A, to be able to invade the population.

Changing the model's parameters, as by increasing the potency of the toxins, would lead to a change in the observed ESS, as shown in Figure 2B. Here, toxin production is the dominant strategy. In the asymmetric aggression scenario for example, while the toxin producer grows slower than the non toxin producer opponent at the earlier stages of growth, toxin accumulation in the system later inhibits the growth of the sensitive strain (around *t* = 18 h) leading to the monopolization of the available resources by strain adopting the toxin production strategy. Through inspecting the two other scenarios as well, the toxin production strategy leads always to a higher fitness regardless of the strategy of the opponent and aggression is favored. However, as expected, when the two strains opt to toxin production they both end up with much lower fitness than the no aggression scenario.

**Figure 3:**
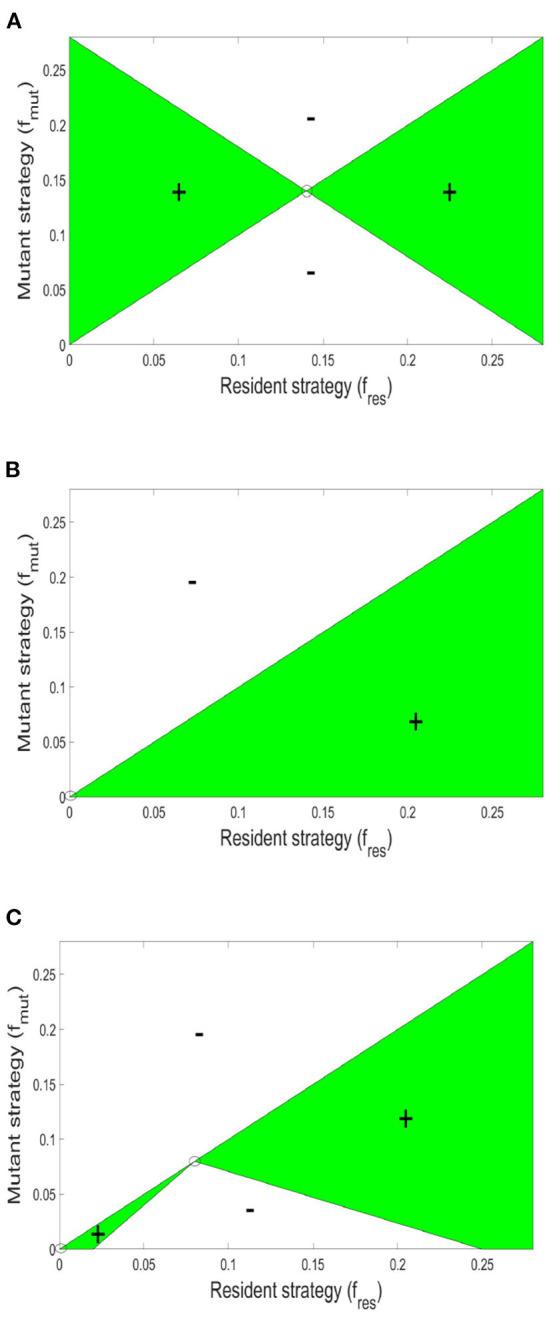
Pairwise invasibility plots for the competition between two constitutive toxin producing strains under different conditions. The green areas are where a mutant toxin production strategy, *f*_*mut*_, fares better than a resident toxin production strategy, *f*_*res*_. And thus can spread in the population. A resident strategy is said to be evolutionary stable if it can not be invaded by any mutant. **(A)** High toxin lethality/high initial nutrient concentration [*N*(*t* = 0) = 10^4^ mg l^−1^, *K*_*T*_ = 15 × 10^−4^ l mg toxin^−1^ h^−1^]: here, producing toxin can be advantageous up to a certain extent. When the two strains are producing toxin at an optimal investment rate f^*^, lying here at the center of the graph, no mutant can fare better. **(B)** At low toxin lethality/ low initial nutrient concentration parameter region [*N*(*t* = 0) = 10^3^ mg l^−1^ K${}_{T}=1.5\times 10^{-4}$ l mg toxin^−1^ h^−1^: in such conditions producing toxin is disadvantageous to the producing strain. Hence in a population in which the strains are attacking each other using an investment in toxin production, *f*_*res*_, any mutant with lower level of toxin production, *f*_*mut*_ < *f*_*res*_, will fare better. The graph shows that the only strategy that is evolutionary stable lies at the origin: when the two strains are not producing toxins at all. **(C)** The pairwise invasibility plot when multiple evolutionary stable growth strategies exist [*N*(*t* = 0) = 10^3^ mg l^−1^, $K_{T}=13\times 10^{-4}$ l mg toxin^−1^ h^−1^]. Here, both no toxin production and toxin production at an optimal investment rate are evolutionary stable.

Afterwards, we allow the fraction of investment in toxin production to vary. To find the specific level of investment in toxin production corresponding to the ESS that should be adopted by both strains at evolutionary equilibrium, an invasion analysis is carried out. For that, it is assumed that the two strains initially have a level of investment in toxin production *f*_1_ = *f*_2_ = *f*_*res*_. And the question is whether a mutant of any of the two strains that has a different level of investment in toxin production, *f*_*mut*_, would be able to invade the population. For that the invasion index of the mutant strategy, calculated according to Equation (7), must be higher than one. By repeating this analysis for all possible combinations of *f*_*mut*_ and *f*_*res*_ (see Figure 3), the value of *f*_*res*_ where no mutant strategy fares better than the resident strategy can be determined. At high toxin lethality/ initial nutrient concentration, the optimal investment in toxin production can be found using the pairwise invasibility plot depicted in Figure 3A where at the optimal point, $f_{1}=f_{2}=f^{*}$, a mutant belonging to any of the two strains adopting a lower or higher toxin investment fraction than f^*^ will achieve lower final biomass when compared to the resident strategy. The optimal strategy in such situation is investment in toxin production, but only up to a certain extent, f^*^, after which, the gain from producing toxin is outweighed by the growth deficiency due to the excessive investment in toxin production at the expense of biomass production. The optimal point, f^*^, will depend on the toxin killing rate and the initial nutrient concentration, among other factors (see Niehus, 2016 for more extensive discussion). By contrast, at low toxin lethality/ initial nutrient concentration, see Figure 3B, for two strains adopting a certain level of investment in toxin production, *f*_1_ = *f*_2_ = *f*_*res*_, whenever a mutant emerges with a lower level of investment, *f*_*mut*_ < *f*_*res*_, it will end up with higher fitness than the resident strategy. The only point where no mutant strategy can gain an improvement in fitness is at the origin, when the resident population produces no toxin, *f*_*res*_ = 0. Hence, the evolutionary stable state of this competition is when the two strains fully commit to peaceful growth.

**Figure 4:**
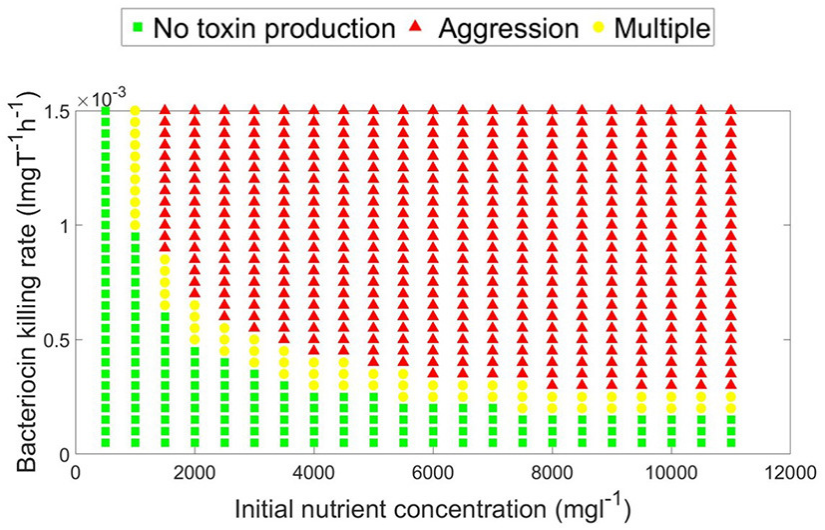
A parameter map showing the optimal, evolutionary stable, growth strategy (aggressive vs. no toxin production) under different combinations of toxin killing rate and initial nutrient concentration. For a constitutive toxin producer, aggression is favored as the initial nutrient concentration and the toxin lethality increase. Peaceful growth conversely is the optimal growth strategy when nutrients are more limited and the toxin is less lethal. A third, narrower, parameter region exists which is characterized by the existence of multiple evolutionary stable growth strategies.

A parameter map showing the *N*_0_ and *K*_*T*_ combinations at which each growth strategy, no toxin production vs. aggression, is dominant is shown in Figure 4. It is noted that two main parameter regions emerge. When the initial nutrient concentration and toxin lethality is low, a strain is better off investing in fast growth. Investing in toxin production on the other hand is most beneficial when the nutrient concentration is higher and the toxin killing rate is relatively high. It is also observed in Figure 4 that as the bacteriocin killing rate increases, aggression becomes the dominant strategy. However, this will lead to grave consequences as the fitness of both contestants deeply suffers from mutual aggression. This goes inline with previous research, where an analysis by Frank (1994) of the competition between a toxin producer and a sensitive strain has shown that investment in toxin production becomes more favorable at high nutrient concentrations and high toxin lethality. Finally, a narrow parameter region exists where both peaceful growth and producing toxin at an optimal level are non-invadable ESS strategies, an example of a pairwise invasibility plot where this occurs is shown in Figure 3C.

It is seen from the well-mixed culture simulations, that aggression is favorable at high initial nutrient concentration and high toxin lethality. In such conditions, the game dynamics can be classified as a Prisoner's dilemma, where each of the two strains is better off engaging in aggression using an optimal level of investment in toxin, f^*^, as shown through the pairwise invasibility plots. However, at equilibrium, when both strains engage in releasing toxins, they end up with much lower final biomass than the peaceful growth scenario. The cost of aggression is rather high in well-mixed bacterial systems.

### 3.2. Competition dynamics in a biofilm

How does the competition dynamics change when moving from a well-mixed system, to a spatial mode of growth, as in biofilm growth? To investigate that, the competition between two strains growing on a surface is simulated using IbM under conditions of high toxin lethality where aggression is a favorable strategy. The simulation is initialized with a randomly distributed layer of cells. Three scenarios are again simulated: (i) two non toxin producing strains growing together, (ii) a toxin producing strain vs. a non producer, and (iii) the case of two toxin producers against each other.

**Figure 5:**
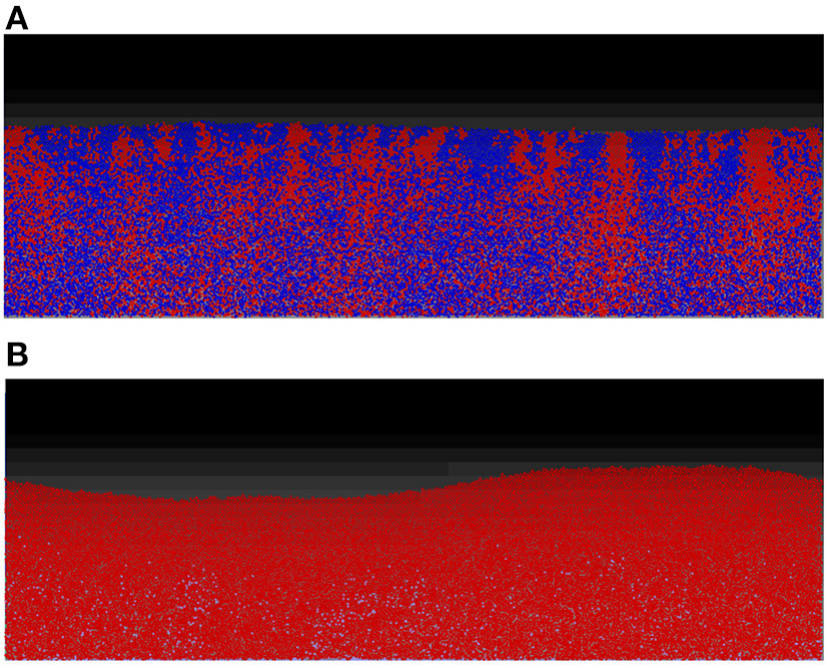
While the spatial mode of growth significantly reduce the cost endured in the mutual aggression scenario due to the resulting spatial segregation, the outcomes of other scenarios are not significantly altered from the well-mixed growth setting. **(A)** No aggression: competition between two non toxin producers in the biofilm mode of growth. Starting from an initial mixed layer of cells, the two strains grow together to form a mixed biofilm with little linage segregation. **(B)** Asymmetric aggression: competition between a toxin producing strain (red) and a non toxin producing strain (blue), in spatial settings. The constitutive toxin producer come to dominate the competition at early stage, leading to the elimination of the non toxin producing strain.

**Figure 6:**
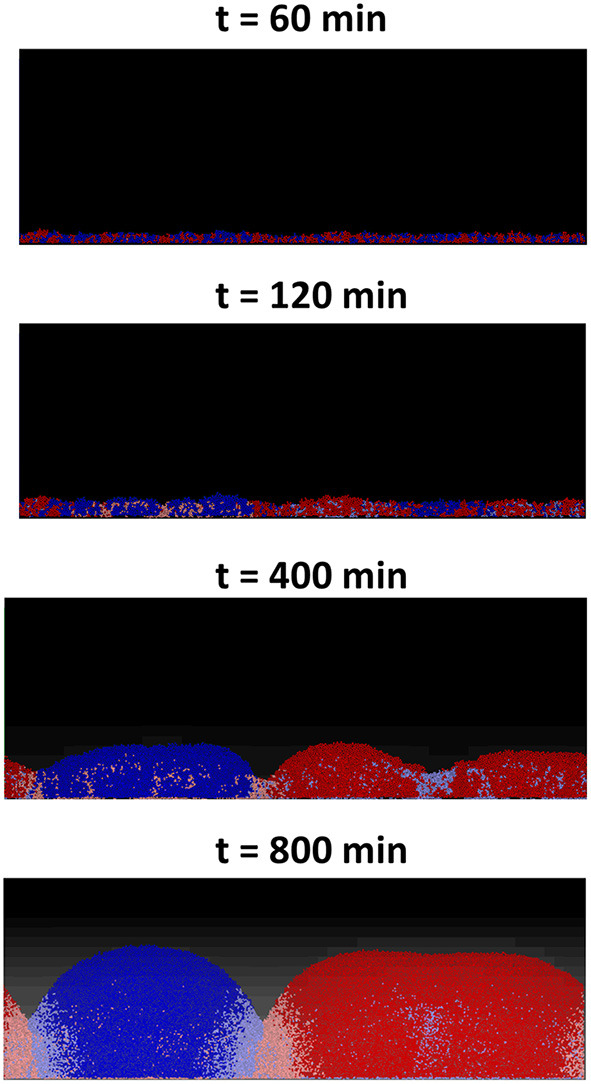
An individual-based model of a competition between two toxin producing strains, growing together as a biofilm starting from an initial mixed layer of cells. The two strains gradually segregate into two distinct clusters. The cells lying at the clusters' boundaries are experiencing negative growth, due to the toxins' effect, and the further a cell lies from the clusters' boundaries, the lower the damage it suffers.

For the first two scenarios, (i) and (ii), shown, respectively, in Figures 5A,B, the results produced are similar to their counterparts in a well-mixed system: the two strains grew to form a mixed biofilm in the first scenario and the toxin producer completely dominates the biofilm in the second. In the case of two toxin producers however (iii), it is observed that the two strains gradually segregated from each other, as seen in Figure 6.

This is an example of pattern formation resulting from local self-activation, growth, and lateral inhibition, due to toxins, as explained in Meinhardt and Gierer (2000). This process of pattern formation is guided by the initial asymmetries present in the initial layer of cells which get amplified by positive feedback loops to result in a significant deviation from the initial near homogeneous state, causing the segregation effect (Magoroh, 2017). The same process can also be explained through the lens of clonal expansion of cells, which allows the segregation of the two strains leading to the formation of the distinct clonal groups/clusters, ultimately reducing the strength of the non-kin competition within the biofilm.

These random variations in the initial density and the growth behavior of the cells of the two strains will break the “spatial symmetry” of the contest, meaning that in some segments of the surface, the red strain will have an advantage over the blue strain while in other segments the opposite will occur. An initial advantage in the density or the growth of one strain over the other would lead to a higher concentration of the toxin produced by the former strain. This in turn will lead to amplifying the said advantage in the next layer as the dominant strain's toxin will inhibit the growth of the less ubiquitous strain. This positive feedback loop finally culminates in the segregation of the two strains into distinct clusters across the surface. A consequence of the spatial segregation is a reduction in concentrations, and consequently the effects of the toxins on the cells which lie far from the “front lines.” It can be observed that there is a discrepancy in the growth of the cells based on their position within the each cluster. While the cells growing close to the center of a cluster experience positive growth, the cells lying at the edge of each cluster suffer from negative growth due to the toxins' inhibiting effect. This in turn leads to a significant increase in the overall fitness of the two strains compared to the corresponding well-mixed growth scenario, as most of the cells in the simulation have less exposure to toxins. Furthermore, another factor that contributes to the spatial segregation between the two strains is the bottlenecking effect and the competition over the nutrient (Nadell et al., 2010). The structuring effect resulting from the competition over the nutrient is however relatively weak compared to that resulting from toxin release, as observed by comparing the degree of spatial segregation in Figures 5A, 6.

**Figure 7:**
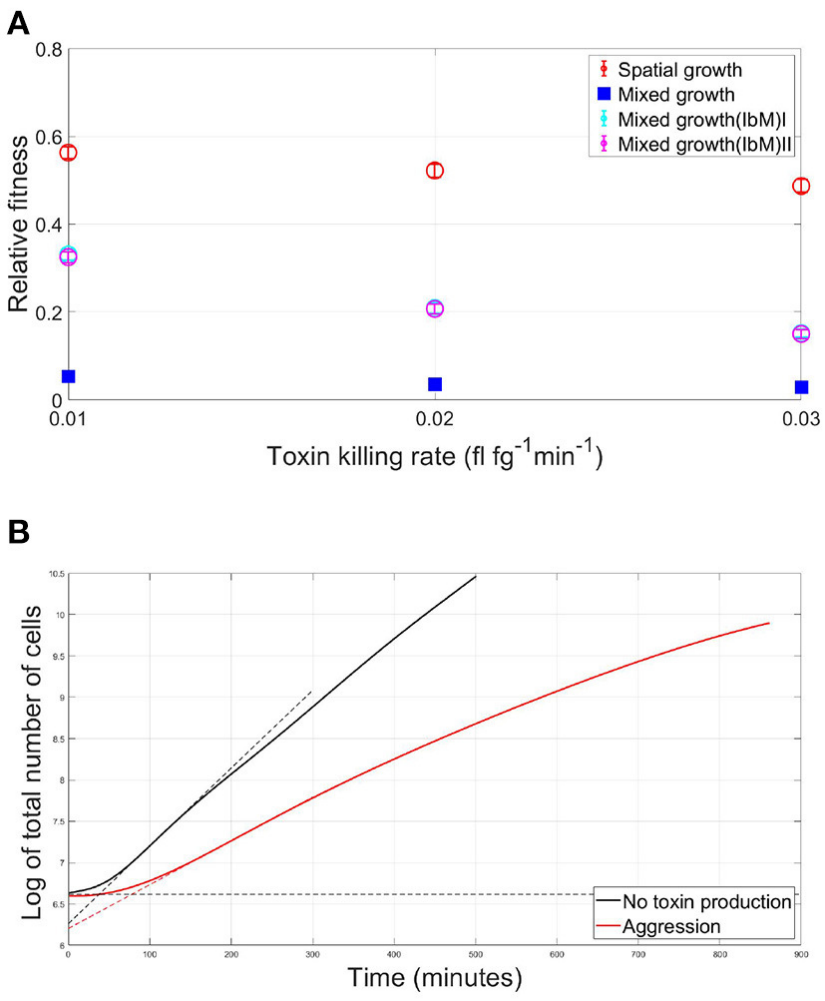
**(A)** The average fitness of two strains engaging in mutual aggression, relative to their fitness in the no aggression scenario, in case of a spatial vs. well-mixed mode of growth, at different toxin lethalities, using three different models of mixed growth as a benchmark. Besides the initial simple mixed growth model, two additional mixed growth models have been created within the IbM environment. In *Mixed growth(IbM)I*, the spatial mixing effect is induced by randomizing the positions of the cells after each iteration, while in *Mixed growth(IbM)II* the concentrations of the toxins are made to be uniform at the horizontal direction by replacing the toxin concentration at each spatial point in each row of the environment grid by the average value of the toxins' concentration in the row to which the spatial point belongs. **(B)** The effect of mutual aggression on the lag phase of a microbial community: the growth curves of the overall population in case of (i) mutual aggression scenario (red) and (ii) No aggression (black) for two strains growing together in a spatial settings as a biofilm. The duration of the lag phase is defined here as the intersection of the maximum slope at the exponential phase with the horizontal asymptote at the initial population level, shown with the dotted lines.

In Figure 7A, the average fitness of a strain in a mutual aggression scenario is plotted as a fraction of the average fitness in case of no aggression, for both the well-mixed and spatial competition settings. For different toxin killing rates, it is noticed that a strain's fitness in case of spatial aggression is consistently much higher than its fitness in case of well-mixed competition. It is crucial to note however that the discrepancy in the fitness of the competing species engaging in aggression between the IbM and the well-mixed simulations can not yet be solely attributed to the spatial structuring effects within the biofilm growth, since there are also other factors that characterize the IbM model which are different from the well-mixed model, namely differences in boundary conditions between the two models: (i) continuous supply of nutrients in the IbM model vs. limited amount of nutrients in the well-mixed model, (ii) toxin leakage from the top boundary of the IbM model while toxins accumulate in the well-mixed model, and finally (iii) stochastic effects only exist in the IbM model. Therefore, to pin down the effects of spatial structuring on the improvement in fitness of the two strains engaging in aggression in a biofilm, two “pseudo” well-mixed models have been created within the IbM environment. In the first model, named *Mixed growth(IbM)I*, the well-mixed growth within an IbM environment is simulated by randomizing the positions of the cells after every iteration, following Schluter et al. (2016). Additionally, a second model, *Mixed growth(IbM)II*, is created where local differences in toxin concentrations across the biofilm is eliminated by uniformizing the toxins' concentrations at the horizontal direction. This is done by replacing the toxins' concentrations at each spatial unit in each row of the environment grid by the average of the toxin's concentration in this row of spatial units. This way, toxins in the simulation keep diffusing away in the vertical direction while being uniform in the horizontal direction across the biofilm, regardless of the local biomass densities of each strain. Results from both models still show a significant improvement in the strains' fitness at the spatially structured IbM when compared to the two pseudo well-mixed models, which both provided similar results. This shows that spatial structure plays a significant role in alleviating the cost of aggression in biofilms.

The growth curve of bacteria is known to start with a lag phase, during which, little to no cellular division occurs, as cells adapt to the new medium by synthesizing necessary enzymes, RNA and other cellular components essential to initiate the exponential phase of growth (Rolfe et al., 2012; Madigan et al., 2018). An interesting observation in the case of competition between two toxin producers is the emergence of a competition-induced lag time in the overall population growth curve. The lag time of the total population in case of mutual aggression is compared to the no toxin production scenario in Figure 7B, where the lag time is defined as the intersection of the maximum slope at the exponential phase with the horizontal asymptote at the initial population level in the graph showing the evolution of the log of the number of cells with time (Baty and Delignette-Muller, 2004). The emerging lag time is associated with the initial, mixed, phase of growth of the population. When the two aggressive strains are fully mixed, they are under the growth inhibiting effect of each other, leading to a phase of slow linear growth. Afterwards, as spatial segregation comes into effect, the two strains became less affected by each other's inhibiting effect. This leads to the beginning of the exponential phase of growth. It should be remarked here that the exponential phase of growth is a consequence of the high substrate diffusivity assumed for this model, which does not allow for the emergence of linear growth rate regime observed in other modeling studies (Ward et al., 2005). It is also observed that the slope during the exponential growth phase decreases with time, this dampening effect is due to the accumulation of the toxin in the medium, leading to a progressively lower slope compared to the no toxin case. Finally, it should be also noted that while the lag time in Figure 7B is determined using the same method as when calculating the classic lag phase in batch cultures, the nature of the lag time here is different as it emerges from the competitive social interaction between the cells, and not due to the individual adaptation of the cell to the environment.

#### 3.2.1. Effect of varying toxins' diffusivities

**Figure 8:**
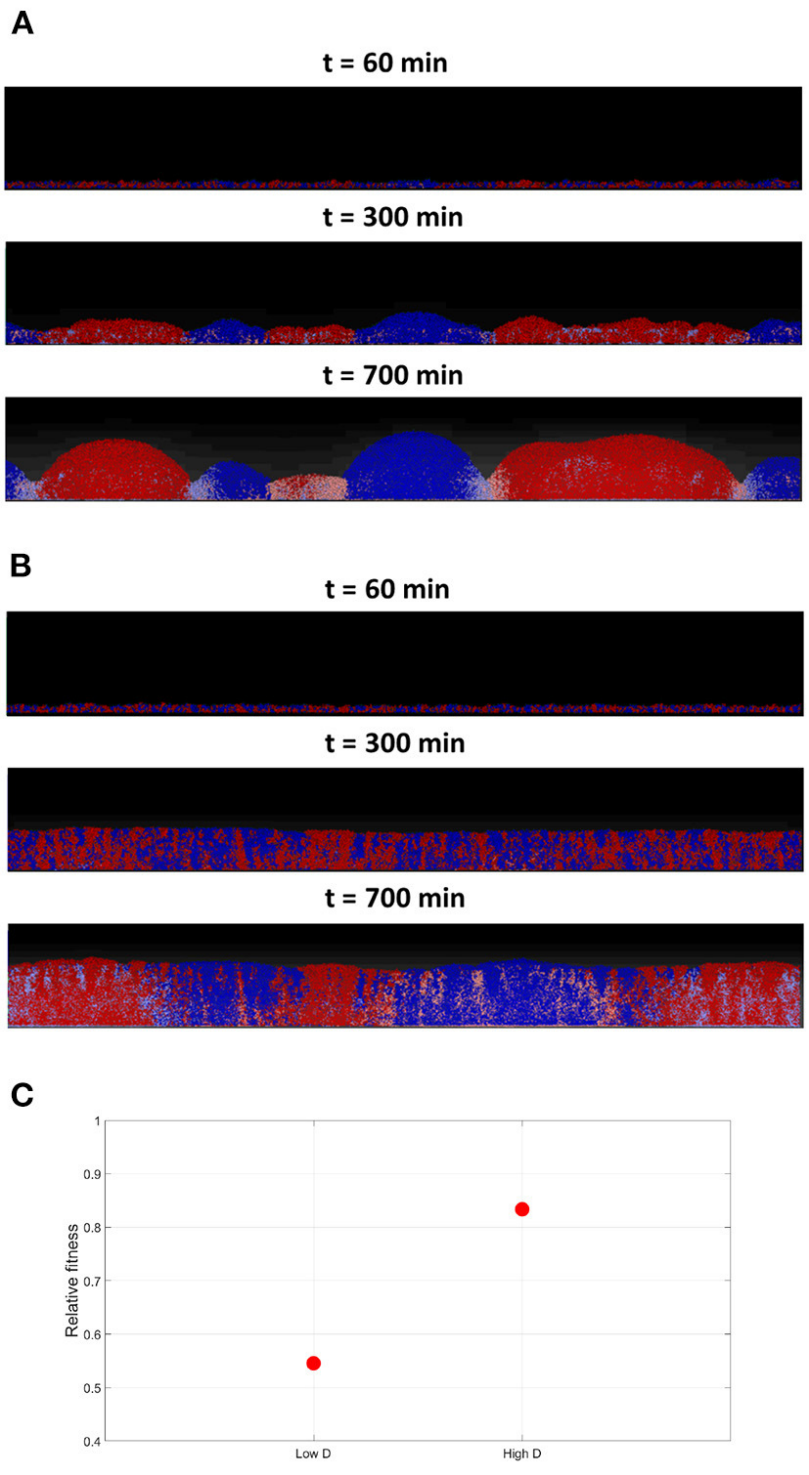
**(A)** The competition between two toxin producing strains at low toxins diffusivities in a grid of 1, 200 × 200μ*m*. The low toxin diffusivity, increases its effectiveness, resulting in clusters with sharp, well-defined, boundaries. **(B)** Competition between two toxin producing strains, at high toxins diffusivities in a grid of 1, 200 × 200μ*m*, where *D*_*T*_ is set to 10 times the nominal value. The high toxin diffusivity reduces its effectiveness as it gets more quickly diluted, resulting in a more uniform growth with less defined boundaries between the two spatially segregated strains. **(C)** The average fitness of the two strains engaging in mutual aggression, relative to their fitness in the no aggression scenario, under low and high diffusivities.

Next, the effect of varying toxins' diffusivity is investigated, by comparing the outcomes of the competition under low and high toxin diffusion coefficients. A toxin's diffusivity is known to be dependent on the medium composition, temperature and the toxin itself. In the low diffusivity scenario, where *K*_*T*_ is set at the nominal value, it is noticed in Figure 8A that segregation happens earlier in the simulation, giving rise to curved clusters with well defined borders. Between the clusters, a no-man's land where bacteria experience negative growth can be located. A higher toxins' diffusivity would be expected to lead to longer wavelength patterns, and thus wider clusters. When running the high toxins' diffusivity scenario, by setting *K*_*T*_ to ten times the nominal value, a more complex picture emerge as seen in Figure 8B; it is observed that the segregation happens later in the simulation, and the growth is more uniform. Also, while the cells growing within each cluster from the opposite strain get inhibited, the effects on the borders are less clear. The reason is that when the toxin's diffusivity increases, its effectiveness decrease, as it gets quickly diluted in the medium. Increasing toxin's diffusivity is here equivalent to decreasing toxin's killing rate, and consequently, its effectiveness. The higher the diffusivity, the more the toxin gets diluted within the biofilm and the lower its concentration. Hence, the lower toxin effectiveness will have two results: a delayed, less sharp, spatial segregation, and a lower inhibiting effect on the growth of the two strains involved, as shown in Figure 8C, when compared to the low diffusivity scenario.

### 3.3. The territorial nature of microbial aggression

Previous research has shown that benefit-to-cost ratio of engaging in toxin production in structured habitats plays a key role in the promotion of evolution of anticompetitor toxins (Chao and Levin, 1981) as well as maintaining microbial diversity (Durrett and Levin, 1997). Chao and Levin (1981) shows that in structured habitats a colicin releasing bacteria is more likely to outgrow a sensitive strain as an inhibition zone is created out the microbes of the colicinogenic colonies which increases the availability of the resources to them, thus increasing the benefit-to-cost ratio of the competition. Our paper highlights the territorial nature of microbial conflicts between mutually aggressive strains and how spatially structured competition helps to increase the benefit-to-cost ratio of the competition by reducing the cost of engaging in fight. This cost encompasses here not only the metabolic cost of producing the toxin, but also the growth inhibiting effects resulting from engaging in microbial warfare with another strain. The reduction in growth rate of two toxin producing strains competing against each other has been shown to be dependent on the mode of growth. In a well-mixed system, the strength of the intra- and inter-species competition is the same. On the other hand, in a spatially structured system, the emergence of clustering reduces the strength of the intra-species competition, allowing the cells from the same kin to enjoy a relatively harm-free growth.

Different strains engage in a fight to establish their own territories at the early phase of biofilm growth. Once segregation happens, most of the cells from each strain will enjoy harm-free growth, away from the front-lines of the war. It is important to remark that spatial segregation can be reinforced by other aspects of competition, other than toxin production, as well. Nutrient limitation and growth bottlenecks have been known to give rise to spatial segregation between different genotypes growing out of a well-mixed, diverse biofilm (Hallatschek and Nelson, 2008; Nadell et al., 2008; Mitri et al., 2016). Furthermore, besides the spatial structuring, other factors could play a role in promoting the evolution of aggression which have not been the focus of this paper, such as the scale of competition (Gardner and West, 2004; Bucci et al., 2011). Additionally, to keep the model simple, only the competition between constitutive toxin producers has been considered. In reality, the use of damage-dependent regulation mechanisms for toxin release is widespread (Breidenstein et al., 2011; Dobson et al., 2012; Cornforth and Foster, 2013; LeRoux et al., 2015), which would have also lead to reducing the cost of aggression in the spatial competition compared to well-mixed growth as the fight would stay confined to the clusters' boundaries, while the rest of the cells can fully invest in growth.

The territorial nature of aggression in the biofilm mode of growth would be expected to increase aggression due to the notable increase in the benefit-to-cost ratio in a territorial conflict compared to the well-mixed competition. It has been well-documented that territorial conflicts have a particularly aggressive nature due to the high benefit-to-cost (Georgiev et al., 2013). This would be especially true in the microbial world as well, where the dominance of a bacterial strain over a territory is translated into a monopoly over the resources there, as well as being relatively safe from the harm of its competitors. Another interesting observation that characterized the spatial simulations is the emergence of a socially-induced lag time in the overall growth curve of the biofilms. This is due to the growth-inhibiting effect of the toxins at the early, mixed, stage of competition. Since the lag phase has been traditionally understood as a result of non-social mechanisms which are related to the adaptation of the individual cells to a new environment, these results can serve to highlight that social interactions can also induce a lag time in the overall population growth curve of a microbial community.

## Data availability statement

The raw data supporting the conclusions of this article will be made available by the authors, without undue reservation.

## Author contributions

Conceptualization, resources, writing, and review: IH and JVI. Investigation and literature review and writing—original draft preparation: IH. All authors contributed to the article and approved the submitted version.

## Funding

This work was supported by the KU Leuven Research Council (OPTEC Center-of-Excellence Optimization in Engineering OPTEC and project C24/18/046), by the ERA-NET FACCESurPlus FLEXIBI Project, co-funded by VLAIO project HBC.2017.0176, by the Fund for Scientific Research-Flanders (projects G.0863.18 and G.0B41.21N), and by the European Union's Horizon 2020 Research and Innovation Programme (Marie Sklodowska-Curie grant agreement numbers 813329 and 956126).

## Conflict of interest

The authors declare that the research was conducted in the absence of any commercial or financial relationships that could be construed as a potential conflict of interest.

## Publisher's note

All claims expressed in this article are solely those of the authors and do not necessarily represent those of their affiliated organizations, or those of the publisher, the editors and the reviewers. Any product that may be evaluated in this article, or claim that may be made by its manufacturer, is not guaranteed or endorsed by the publisher.
